# Assessing and adjusting for bias in ecological analysis using multiple sample datasets

**DOI:** 10.1186/s12874-025-02552-y

**Published:** 2025-04-24

**Authors:** Qingfeng Li

**Affiliations:** Department of International Health, Johns Hopkins Bloomberg School of Public Health, 615 North Wolfe Street, E-8136, Baltimore, MD 21205 USA

**Keywords:** Ecological analysis, Causality, Causal analysis, Aggregate measures, Sample datasets, Sampling fraction bias, Measurement error

## Abstract

**Background:**

Ecological analysis utilizes group-level aggregate measures to investigate the complex relationships between individuals or groups and their environment. Despite its extensive applications across various disciplines, this approach remains susceptible to several biases, including ecological fallacy.

**Methods:**

Our study identified another significant source of bias in ecological analysis when using multiple sample datasets, a common practice in fields such as public health and medical research. We show this bias is proportional to the sampling fraction used during data collection. We propose two adjustment methods to address this bias: one that directly accounts for the sampling fraction and another based on measurement error models. The effectiveness of these adjustments is evaluated through formal mathematical derivations, simulations, and empirical analysis using data from the 2014 Kenya Demographic and Health Survey.

**Results:**

Our findings reveal that the sampling fraction bias can lead to significant underestimation of true relationships when using aggregate measures from multiple sample datasets. Both adjustment methods effectively mitigate this bias, with the measurement-error-adjusted estimator showing particular robustness in real-world applications. The results highlight the importance of accounting for sampling fraction bias in ecological analyses to ensure accurate inference.

**Conclusion:**

Beyond the ecological fallacy uncovered by Robinson’s seminar work, our research identified another critical bias in ecological analysis that is likely just as prevalent and consequential. The proposed adjustment methods provide potential tools for researchers to adjust for this bias, thereby improving the validity of ecological inferences. This study underscores the need for caution when pooling aggregate measures from multiple sample datasets and offers potential solutions to enhance the reliability of ecological analyses in various research domains.

**Clinical trial number:**

Not applicable.

## Introduction

Ecological analysis is frequently conducted in academic research, though causality ultimately operates at the individual level [1]. Personal and environmental factors determine an individual’s outcome. Associations observed at group or population levels are essentially summaries of causal effects on individuals within the population. For example, vaccination improves a child’s immunity to diseases; similarly, at the ecological level, children in regions with higher vaccination coverage exhibit stronger immunity. The concept that causality operates at the individual level implies that causality does not directly manifest at the population level but it does not exclude the influence of environmental factors. Even though causality is typically discussed in individual contexts, environmental and social phenomena frequently involve causality at group or ecological levels. Group characteristics can impact an individual outcome, a phenomenon referred to as the group effect in sociology [2, 3]. 

Ideally, inferring individual-level causality requires individual-level data that links an individual’s explanatory variables to their outcomes. However, ecological analyses that attempt to make individual-level inferences using ecological data are common in practice for several reasons. First, individual-level data linking individuals’ exposure and outcome measures are often unavailable [4]. Second, for studies of rare events or diseases, aggregate data may offer higher accuracy than individual-level data. Third, certain research questions can only be addressed using ecological inference technique [5]. Even research questions concerning aggregates may be better addressed if a valid individual-level inference can be drawn from the data [5]. It is important to note that in this paper, “ecological inference” refers to the process of drawing individual-level conclusions using aggregate data, although in some literature, the term also refers to drawing ecology-level inference from aggregate data [6]. 

Ecological inference methods have been extensively used in political science, public health, geography, sociology, economics, and history for well over a century [7]. Particularly in the last two decades, there has been a significant increase in ecological inference studies [8]. A study cataloged five pages of ecological studies to illustrate the diversity of topics where ecological inference has been applied [5]. Ecological studies continue to be an active field of research, with recent significant advancements in both methodologies and applications [9, 10]. Innovations in computational and statistical methodologies have enhanced the analysis of complex ecological data structures, facilitating more nuanced insights into ecological phenomena [11]. These methodological developments have been applied across various domains [12–15]. 

Researchers have long debated the merits and drawbacks of making individual-level inferences using aggregate data, especially following Robinson’s warning about the dangers of ecological fallacy [16]. The ecological fallacy, considered one of the most extended problems in ecological studies and a major limitation of ecological inference, occurs when conclusions drawn from ecological data are erroneously applied to individuals within those groups [5, 7]. This fallacy stems from the assumption that individuals in the group have the average characteristics of the group [17]. Robinson concluded that while individual-level and ecological correlations can coincide under certain assumptions, the only reasonable assumption in practice is that ecological correlations differ from the underlying individual-level associations. Duncan et al. later expanded the concept of ecological fallacy to include regression coefficients in linear models [18]. Numerous papers and books have since discussed the sources, consequences, and attempted solutions to the ecological fallacy [17, 19–21]. The development of adjustment approaches to obtain correct individual-level correlations from ecological data has been a central focus of ecological inference research [22]. 

Many assumptions have been proposed to mitigate ecological bias in ecological studies. Researchers have sought to make the weakest possible assumptions while ensuring the validity of their inferences. This paper makes a relatively strong assumption that individuals are independent of each other, meaning that a group is merely a collection of individuals. This assumption of independent individuals is fundamental in statistical analysis, as it simplifies both modeling and the result interpretation [23, 24]. This assumption underlies many statistical tests and models, including t-tests, ANOVA, and classic regression analysis [25]. When individual observations are independent, the mathematical derivation of estimators and their statistical properties becomes more straightforward [26]. There have also been numerous studies to assess and address violations of the independence assumption in practice [27, 28]. It is important to emphasize that the assumption of independent individuals does not eliminate the risk of ecological fallacy. Various factors, including reduced variability within groups, the emergence of spurious patterns due to aggregation, and omitted variable bias at the group level, can still lead to ecological fallacy under the independence assumption [29, 30]. 

## Methods

The existing literature on ecological studies and the associated fallacy predominantly focuses on population data, even though sample data is more prevalent in academic research [31]. This section uses formal mathematical expressions to demonstrate the bias that arises when aggregate measures from multiple surveys are combined; this bias exists even under the strong assumption of independent individuals. We begin the statistical discussion by defining notations.

- $\:c$ indexes groups in ecological studies, which may represent cities, countries, or cohorts.
- $\:{N}_{c}$: the number of individuals in group $\:c$ in the population
- $\:s{f}_{x}$: the sampling fraction (the ratio of sample size to population size) for the survey that collects variable $\:x$
- $\:s{f}_{y}$: the sampling fraction for the survey that collects variable $\:y$
- $\:{n}_{xc}$: the number of individuals in group $\:c$ in the survey that collects $\:x$, where $\:{n}_{xc}={N}_{c}*s{f}_{x}$
- $\:{n}_{yc}$: the number of individuals in group $\:c$ in the survey that collects $\:y$, where $\:{n}_{yc}={N}_{c}*s{f}_{y}$
- $\:{x}_{c}$: the average value of $\:x$ for group $\:c$ in the sample, defined as $\:{x}_{c}\equiv\:\frac{1}{{n}_{xc}}{\sum\:}_{i\in\:c}{x}_{i}$
- $\:{y}_{c}$: the average value of $\:y$ for group $\:c$ in the sample, defined as $\:{y}_{c}\equiv\:\frac{1}{{n}_{yc}}{\sum\:}_{i\in\:c}{y}_{i}$

Ecological analysis is commonly employed when exposure and outcome measures are derived from different sources. For instance, ecological analysis is unnecessary when both vaccination history and health measures are available for individual children. In that case, individual-level modeling would be more appropriate. However, ecological analysis becomes essential when group-level aggregate measures must be linked across separate sources, such as vaccination history from a sample survey and health metrics from civil registration data. Consequently, this report focuses exclusively on scenarios where exposure and outcome measures are collected through separate surveys. When referencing the averages of $\:x$ and $\:y$ in sample data, this study always assumed that these averages are derived from separate samples. To further simplify the discussion, we only consider samples drawn using a simple random selection (SRS) approach. Our conclusions can be readily generalized to more advanced modeling options and data drawn using complex sampling approaches. For two independent SRS surveys, respectively on $\:x$ and $\:y$, the number of individuals from group $\:c$ appearing in both surveys is $\:{N}_{c}*s{f}_{x}*s{f}_{y}$.

### Covariance and correlation coefficient

The covariance between sample group averages of $\:x$ and $\:y$ is

1 $$\begin{array}{l}\:cov\left({x}_{c},{y}_{c}\right)=cov\left(\frac{1}{{n}_{xc}}{\sum\:}_{i\in\:c}{x}_{i},\frac{1}{{n}_{yc}}{\sum\:}_{i\in\:c}{y}_{i}\right)\\=\frac{1}{{n}_{xc}}\frac{1}{{n}_{yc}}\left(\left({\sum\:}_{i=j}cov\left({x}_{i},{y}_{j}\right)\right)+\left({\sum\:}_{i\ne\:j}cov\left({x}_{i},{y}_{j}\right)\right)\right)\end{array}$$

Under the assumption of independent individuals, we have $\:cov\left({x}_{i},{y}_{j}\right)=0$ for $\:i\ne\:j$, which simplifies Eq. (1) to $\:cov\left({x}_{c},{y}_{c}\right)=\frac{1}{{N}_{c}}cov({x}_{i},{y}_{j})$. For the correlation coefficient, we similarly have

2 $$\begin{array}{l}\:cor\left({x}_{c},{y}_{c}\right)=\frac{cov\left({x}_{c},{y}_{c}\right)}{\sqrt{{var(x}_{c})}\sqrt{{var(y}_{c})}}\\=\frac{\frac{1}{{N}_{c}}cov({x}_{i},{y}_{j})}{\frac{1}{{N}_{c}*\sqrt{s{f}_{x}*s{f}_{y}}}\sqrt{var\left({x}_{i}\right)}\sqrt{{var(y}_{i})}}=\sqrt{s{f}_{x}*s{f}_{y}}*cor({x}_{i},{y}_{i})\end{array}$$

This discrepancy between individual-level and ecological-level correlations has been observed in previous studies [32]. In contrast, if $\:x$ and $\:y$ come from the same survey with a sampling fraction of $\:sf$, then $\:cov\left({x}_{c},{y}_{c}\right)=\frac{1}{{N}_{c}*sf}cov({x}_{i},{y}_{j})$ and $\:cor\left({x}_{c},{y}_{c}\right)=cor({x}_{i},{y}_{i})$. In this case, the sampling process affects the covariance but not the correlation coefficient.

### Bias in ecological analysis using multiple sample datasets

A typical ecological analysis involves matching aggregate measures from multiple surveys. Consider a simple linear relationship: $\:{y}_{i}=\alpha\:+{\beta\:}^{i}{x}_{i}+{\epsilon\:}_{i}$, where $\:{\beta\:}^{i}$ denotes the individual-level causality between the exposure and outcome; the superscript $\:i$ indicates the parameter pertains to the individual level, while the subscript $\:i$ is used to index individuals; $\:cov\left[{\epsilon\:}_{i},\:{x}_{i}\right]=0$. Summing the individual equations and dividing by the total number of individuals in each group gives the regression model for group means, $\:{y}_{c}={\alpha\:}_{0}+{\beta\:}^{c}{x}_{c}+{\epsilon\:}_{c}$, where the superscript $\:c$ indicates the parameter $\:{\beta\:}^{c}$ denotes the causality at the ecological level. The assumption of independent individuals implies that $\:cov\left[{\epsilon\:}_{c},\:{x}_{c}\right]=0$. The coefficient in a simple linear regression using group averages of $\:x$ and $\:y$ collected in different sample surveys is,

3 $$\:{\beta\:}^{c}=\frac{cov({x}_{c},\:{y}_{c})}{var\left({x}_{c}\right)}=\frac{\frac{1}{{N}_{c}}cov({x}_{i},{y}_{j})\:}{\frac{var\left({x}_{i}\right)}{{N}_{c}*s{f}_{x}}}=s{f}_{x}\frac{cov\left({x}_{i},{y}_{i}\right)}{var\left({x}_{i}\right)}=s{f}_{x}*{\beta\:}^{i}$$

The individual- and ecological-level regression coefficients differ by a factor of $\:s{f}_{x}$. This discrepancy between $\:{\beta\:}^{i}$ and $\:{\beta\:}^{c}$ occurs because individuals selected in one survey are not necessarily selected in another. As long as the sampling fraction is less than one, this discrepancy is present, and we refer to it as the sampling fraction bias. It should be noted that this simple relationship between individual- and group-level coefficients assumes that the variance and covariance of the aggregate data follow the same relationship as the individual data.

### Adjustment estimator of regression coefficient − 1: inverse-sampling-fraction estimator

Equation 3 suggests an adjustment approach for estimating the individual-level relationship using aggregate data:

$$\:\widehat{{\beta\:}_{ISB}^{c}}=\frac{\widehat{{\beta\:}^{c}}}{s{f}_{x}}$$

where $\:\widehat{{\beta\:}^{c}}$ is the ordinary least squares (OLS) estimator of $\:{\beta\:}^{c}$. We call this the inverse-sampling-fraction (ISB) estimator. According to the Gauss-Markov theorem, $\:\widehat{{\beta\:}^{c}}$ is the minimum-variance linear unbiased estimator (BLUE) of $\:{\beta\:}^{c}$ [33]. Furthermore, if $\:{\epsilon\:}_{c}$ has a normal distribution, as is commonly assumed, $\:\widehat{{\beta\:}^{c}}$ is also the maximum likelihood estimator (MLE). Given that the MLE is asymptotically efficient under regularity conditions, $\:\widehat{{\beta\:}^{c}}$ is therefore asymptotically efficient as well [33]. In summary, $\:\widehat{{\beta\:}^{c}}$ represents the most efficient estimator among linear, unbiased, consistent and asymptotically normally distributed estimators of $\:{\beta\:}^{c}$. The disturbance term is commonly assumed to be normally distributed for mathematical convenience since that assumption facilitates the derivations of statistical properties for the estimators [34, 35]. The implication of violating the assumption depends on the types of violations and the sample size. If the sample size is large enough, the central limit theorem mitigates the impact of non-normality. For small samples, there are many correction approaches, including variable transformations and generalized linear models [36]. 

Applying the Gauss-Markov theorem again, we find that the most efficient estimator of $\:{\beta\:}^{i}$ within the assumed class of estimators is $\:\frac{\widehat{{\beta\:}^{c}}}{s{f}_{x}}$, with a variance of $\:\frac{var\left(\widehat{{\beta\:}^{c}}\right)}{s{f}_{x}^{2}}$. Although this is the most efficient estimator among the assumed class, the variance may become very large when the sampling fraction is small. It should be noted that an increased variance here refers to the precision rather than the relative efficiency of the estimator.

### Adjustment estimator of regression coefficient − 2: measurement-error-adjusted estimator

Deaton proposed an approach that treats sample group means as error-prone estimates of population group means, with the difference between sample and population group means characterized as measurement errors [37]. Following this approach, we apply the results from measurement error models to estimate the ecological model [37]. In our context, we define

$$\:{y}_{c}={y}_{c}^{*}+{v}_{c}$$

$$\:{x}_{c}={x}_{c}^{*}+{u}_{c}$$

where $\:{y}_{c}^{*}$ and $\:{x}_{c}^{*}$ are the population group means; $\:{u}_{c}$ and $\:{v}_{c}$ represent the measurement errors of the sample group means. This is a classical measurement error model, where the observed value represents a noisy version of the true value, which is defined as the population-level value in our study [38]. A direct replication of Fuller’s results yields the following estimator: [39]

$$\:\widehat{{\beta\:}_{MEA}^{c}}={\left({M}_{xx}-{{\Sigma\:}}_{uu}\right)}^{-1}\left({m}_{xy}-{\sigma\:}_{uv}\right)$$

where $\:{M}_{xx}$ is sample moment of independent variables; $\:{m}_{xy}$ is the cross-product matrix of independent and dependent variables; $\:{{\Sigma\:}}_{uu}$ is the sample moment of the measurement errors in independent variables; $\:{\sigma\:}_{uv}$ is the cross-product matrix of measurement errors in the independent and dependent variables. We will refer to this as the measurement-error-adjusted (MEA) estimator.

In his pseudo-panel models, Deaton suggested that all the relevant statistics could be estimated from the microdata, although he did not provide specific methods for doing so. Subsequent studies following Deaton’s approach have also not offered formulas for estimating $\:{{\Sigma\:}}_{uu}$ and $\:{\sigma\:}_{uv}$ from sample data. As the primary focus of this paper is on the concept of sampling fraction bias, we leave the task of developing these estimation methods to future research.

### Simulations

We conducted simulations to illustrate the sampling fraction bias in a simplified setting. Suppose that a single independent variable determines a dependent variable, and individuals are clustered into groups. The data generation model includes group-level random effects and a random error term. The multilevel data structure is given by:

$$\:{y}_{ic}\sim\:N(\alpha\:+{\beta\:}^{c}{x}_{ic}+{b}_{c},\:{\sigma\:}^{2})$$

where.

- $\:{y}_{ic}$ and $\:{x}_{ic}$ are the outcome and exposure, respectively, for individual $\:i$ in group $\:c$;
- $\:{b}_{c}$ is the random effect for group $\:c$

Below is the parameter distributions used to generate the data.

- $$\:{b}_{c}\sim\:N\left(0,{\sigma\:}_{b}^{2}\right)$$
- $$\:\sigma\:\sim\:half-cauchy\left(\text{0,10}\right)$$
- $$\:{\sigma\:}_{b}\sim\:half-cauchy\left(\text{0,10}\right)$$

$\:{\beta\:}^{c}=2$ is used to simplify subsequent comparisons. We simulated 200 groups, with the number of individuals in each group drawn from a uniform distribution $\:U(20000,\:30000)$. The inclusion of group-level random effects introduces clustering effects, which are commonly observed in real-world data. Individuals are independent of each other when $\:{\sigma\:}_{b}=0$, i.e., when clustering effects are absent. Our main conclusions apply to cases with or without clustering effects.

To simulate a typical ecological analysis, we randomly sampled individuals to calculate the average $\:x$ by group and another random sample to calculate average $\:y$ by group. The averages of $\:x$ and $\:y$ are then matched at the group level. For comparison, we also calculated group averages of $\:x$ and $\:y$ from the population data. Estimates from the individual data and population averages serve as the ground truth, against which estimates using sample averages are compared.

The ISF estimator is straightforward to calculate. In contrast, no known method exists in real-world studies to calculate $\:{{\Sigma\:}}_{uu}$ and $\:{\sigma\:}_{uv}\:$in the MEA estimator from the sample data. A challenge lies in the fact that these components involve parameters that must be estimated using population data. However, the calculation is feasible in simulations since the population data is available. $\:{{\Sigma\:}}_{uu}$ and $\:{\sigma\:}_{uv}$ are calculated from the difference between sample and population group averages.

## Results using simulated data

**Fig. 1:**
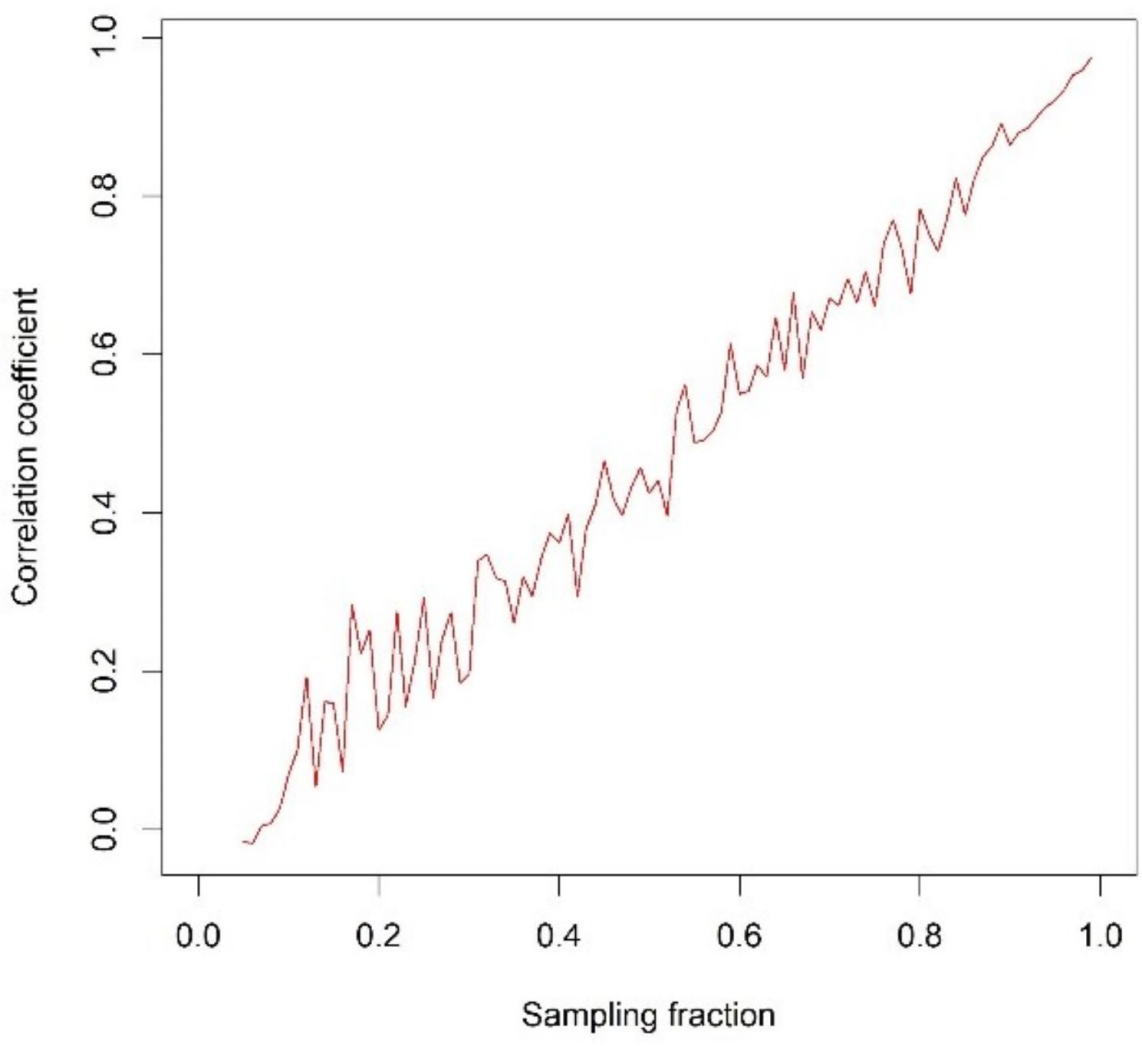
Correlation coefficients between sample averages $\:\varvec{x}$ and $\:\varvec{y}$ by sampling fraction

Figure 1 shows the correlation coefficient in the sample data for sampling fractions ranging from 5 to 99%. As suggested by Eq. (2), the correlation coefficient between group means in the sample data is linearly related to the sampling fraction, with some noise introduced by the data generation and sampling processes.

**Fig. 2:**
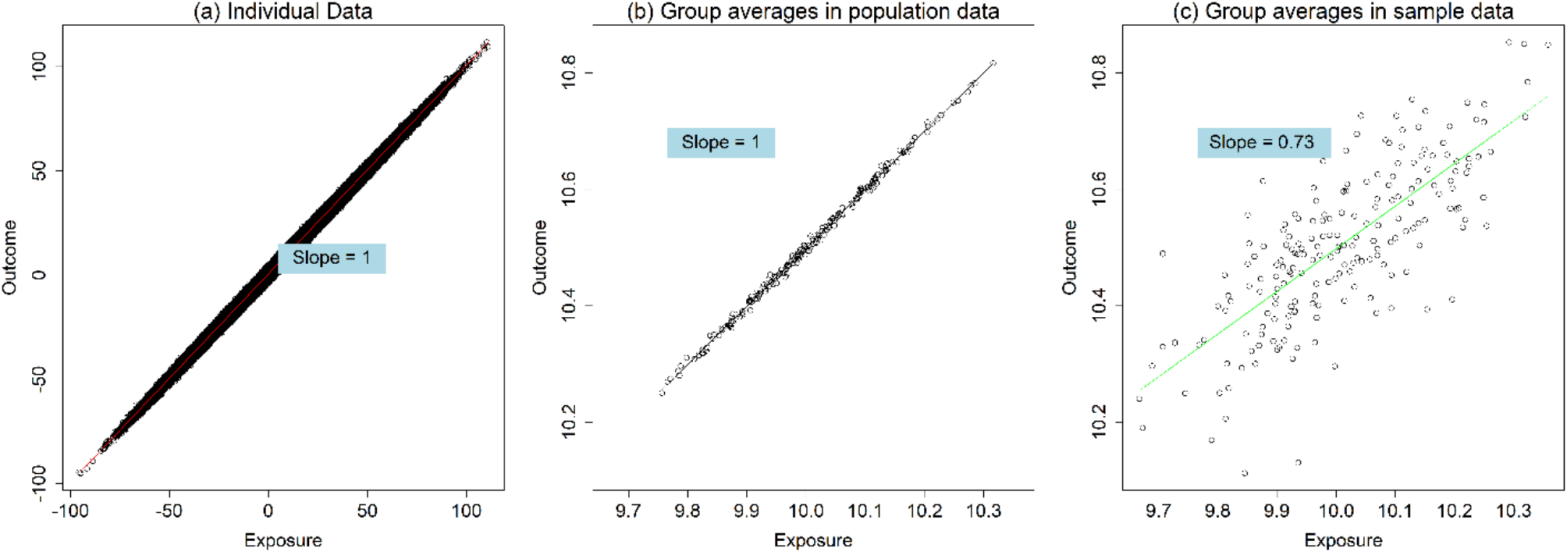
Regression results from individual data, group averages in population data, and group averages in sample data. **a**, Individual data; **b**, Group averages in population data; **c**, Group averages in sample data

Figure 2 presents the regression results derived from individual data, population averages, and sample averages with a sampling fraction of 75%. The slope in the individual population data is 1.00 (95% confidence interval: 1.00–1.00), indicating true individual-level causality. The estimated slope using group averages in the population data is also 1.00 (95% CI: 0.99–1.01). However, the estimated slope estimated using group averages from samples is 0.73 (95% CI: 0.64–0.82), which is smaller than the slopes in the individual data and population averages. This value is close to the sampling fraction of 0.75, as Eq. (2) suggested.

**Fig. 3:**
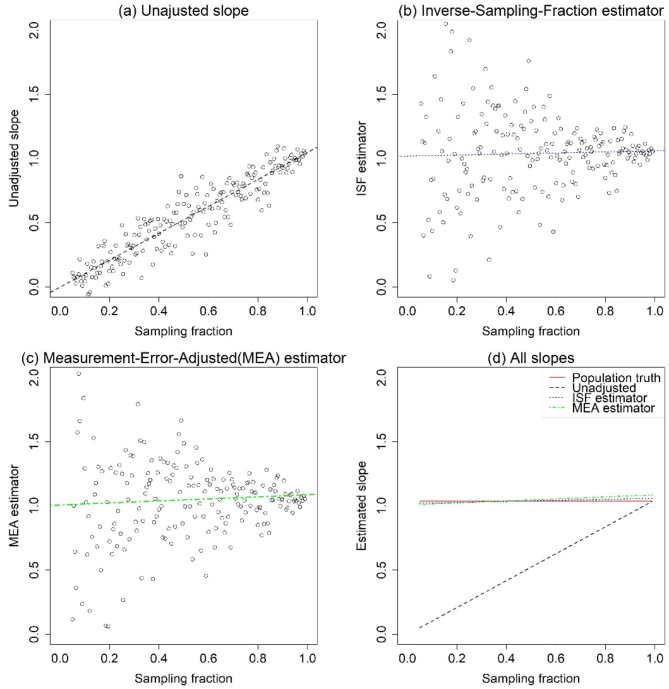
Unadjusted and adjusted slope estimates by sampling fraction; **a**, Unadjusted slope; **b**, Inverse-Sampling-Fraction estimator; **c**, Measurement-Error-Adjusted (MEA) estimator; **d**, All slopes

To explore the relationship between the estimated slope and sampling fraction, we repeated the calculations from Fig. 2 across sampling fractions ranging from 5 to 99%. The results are presented in Fig. 3., where plot (a) illustrates the estimated slopes of the model as a function of the sampling fraction. A diagonal relationship (spanning from 0 to 1 on both axes) is visible, as predicted by Eq. (3). Plot (d) shows all unadjusted and adjusted estimates. The solid red line represents the population-level true value, serving as the gold standard. Both the ISF and MEA estimators perform well, with their fitted curves closely approximating the population-level line.

## Results using Kenya 2014 DHS data

To further illustrate sampling fraction bias in a real-world context, we applied our methods to data from the 2014 Kenya Demographic and Health Survey (DHS). The Kenya 2014 DHS provides a rare opportunity to investigate this bias with a large, well-powered sample that allows for reliable subnational estimates of demographic and health indicators. The survey, conducted between May and October 2014, included 31,709 women aged 15–49 years from 36,430 households across the nation’s 47 counties. Based on a multi-stage stratified survey strategy, the survey was designed to generate representative estimates for most indicators at the regional level and for selected indicators at the county level. It provides critical insights into population trends and health disparities, serving as a vital resource for policymakers, researchers, and program planners to guide evidence-based decision-making. The dataset, which is freely available on the DHS website, has been extensively utilized in numerous studies [40–42]. 

For illustrative purposes, we examined the relationship between women’s parity and the duration of cohabitation with county as the aggregate level for the ecological analysis. While this may not be a particularly compelling research question, it suffices to demonstrate the bias under investigation. Ideally, we would prefer to address a significant research question using real population-level data to assess the magnitude of sampling fraction bias in a real-world setting and evaluate the effectiveness of the adjustment methods proposed in this study. However, due to the unavailability of high-quality data meeting these criteria, we leave that illustration for future research.

**Fig. 4:**
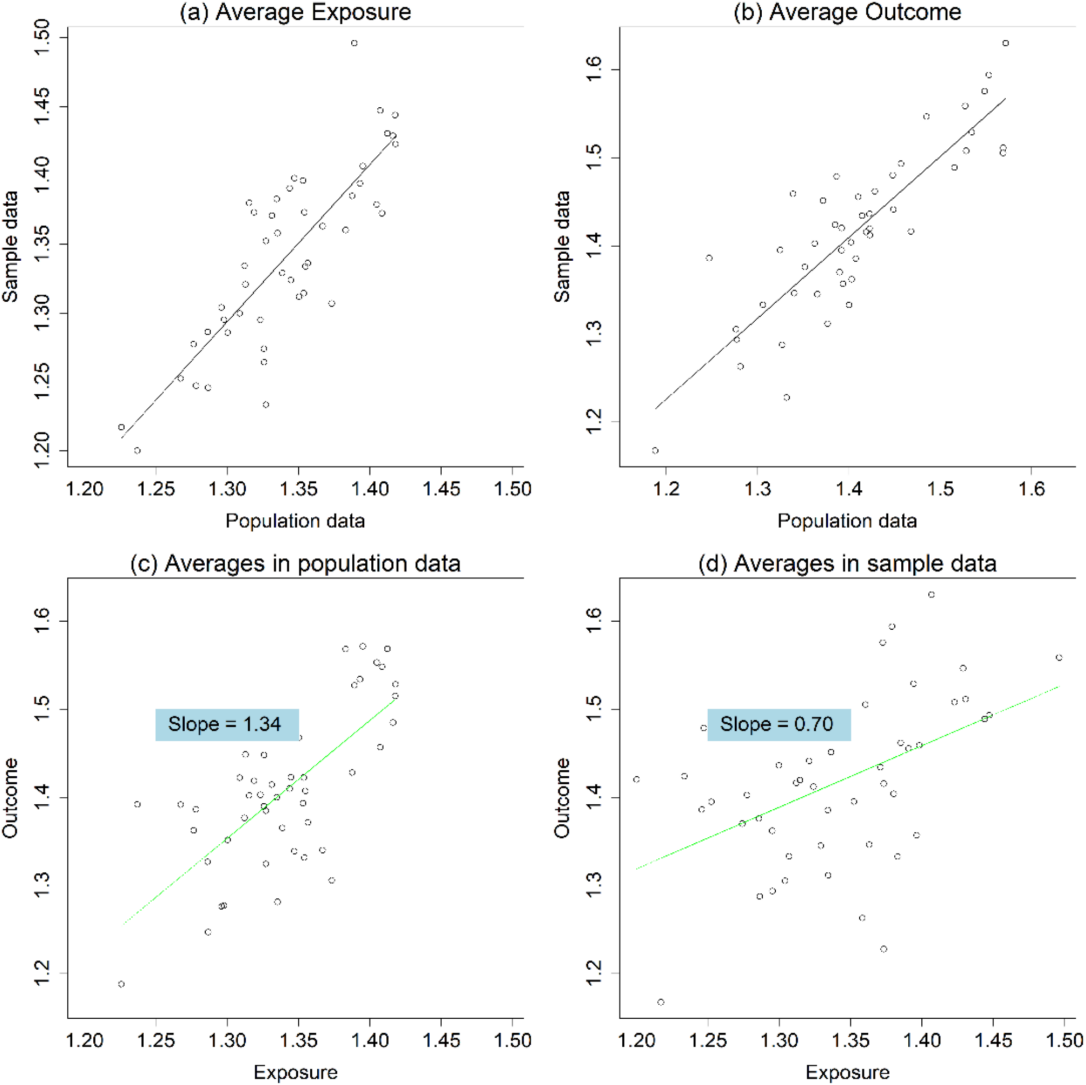
Average exposure and outcome in sample and population data using Kenya DHS 2014. **a**, Average Exposure; **b**, Average Outcome; **c**, Averages in population data; **d**, Averages in sample data.

Although the Kenya 2014 DHS is a survey dataset, it is treated as the population in this analysis. In Fig. 4, sample estimates of group averages closely align with their population counterparts. A positive relationship between sampling fractions and estimated slopes is evident. The estimated slope of linear regression on population averages is 1.34, with an adjusted R-squared of 0.50, indicating an acceptable model fit. This population-level estimate is considered the true ecological correlation, which, under certain conditions, should match the individual-level relationship. However, when using averages from 20% samples, the coefficient dramatically decreases to 0.7, which deviates from the prediction of Eq. (2), likely because individuals in this real dataset are not independent.

**Fig. 5:**
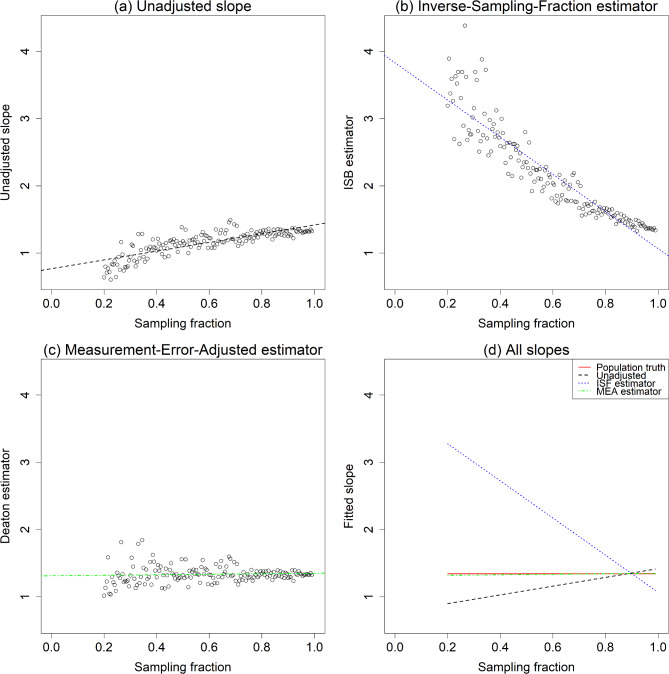
**a**, Unadjusted slope; **b**, Inverse-Sampling-Fraction estimator; **c**, Measurement-Error-Adjusted estimator; **d**, All slopes

Figure 5 shows the estimated slope using sample data across sampling fractions ranging from 20 to 99%. Sampling fractions below 20% yielded too few observations to be useful. Nearly all observations from the simulated data still hold true, though with greater noise, likely due to fitting a simple linear regression model to a multilevel dataset. However, in this real data, the ISF estimator tends to over-adjust for sampling fractions, while the MEA estimator remains valid and stable. Thus, as demonstrated in the simulation, the MEA estimator effectively eliminates bias in sample data estimates.

## Discussion

Ecological analyses often utilize aggregate measures from multiple sample datasets [31, 43]. This paper demonstrates that results derived from such ecological analyses can be biased. This sampling fraction bias may be widespread. The derivations and simulations in this paper assume that samples are drawn using a simple random sampling (SRS) method. However, given that it is the less-than-one selection probability that results in an attenuated correlation between dependent and independent variables, aggregate measures from samples drawn using other sampling methods may also be subject to this bias. Similarly, underreporting in data collection systems, such as incomplete vital registration, can also lead to sampling fraction bias. Different indicators are collected by different systems, each with its own reasons for underreporting. As a result, individuals covered in one system may not be covered in another.

This study focuses on continuous variables, while discrete variables such as binary and categorical variables are also often used in academic research. All binary or categorical variables can be considered as truncated values of underlying latent continuous variables. The relationship between parameters in the latent regression on the latent continuous variables and parameters in regression (e.g., logistic regression) using the truncated discrete variables is well established [33]. Since latent regressions, a type of linear regressions discussed here, are affected by sampling fraction bias, regressions on discrete dependent variables cannot be immune to the sampling fraction bias.

While this paper focuses on cross-sectional models, sampling fraction bias may also be present in longitudinal models. The most widely used estimator in longitudinal models, the fixed-effects estimator, is essentially the ordinary least square estimator (OLS) applied to transformed data [44]. Therefore, the adjustment methods proposed here also apply to fixed effects estimators.

Though this paper uses OLS estimators to illustrate sampling fraction bias, other standard estimators may also be affected. For example, in linear regression models, under the conventional assumption that the disturbance term follows an independent, identically distributed normal distribution, the maximum likelihood estimator (MLE) and OLS estimators are equivalent. As a result, maximum likelihood estimates may also be biased by the sampling fraction when using aggregate measures from sample data.

## Conclusion

In his seminal work, Robinson highlighted the dangers of the ecological fallacy and urged researchers to exercise caution when drawing individual-level inferences from aggregate data. Similarly, this study underscores the need for vigilance when pooling aggregate measures from multiple sample datasets. The sampling fraction bias identified here is another critical issue that must be addressed to ensure the validity of ecological inferences. By proposing adjustment methods and demonstrating their effectiveness through simulations and real-world data, this study contributes to the ongoing efforts to improve the accuracy and reliability of ecological analyses.

In conclusion, the sampling fraction bias represents a significant challenge in ecological research, when combining data from multiple sources. While the proposed adjustment methods offer promising solutions, further research is needed to refine their implementation and expand their applicability. By addressing this bias, researchers can enhance the validity of their findings and contribute to more accurate and actionable insights in fields such as public health, sociology, and economics. This study serves as a call to action for the research community to develop and adopt methods that account for sampling fraction bias, ensuring that ecological analyses remain a robust tool for understanding complex relationships using population-level data.

## Data Availability

No datasets were generated or analysed during the current study.
